# A systematic analysis of the phloem protein 2 (PP2) proteins in *Gossypium hirsutum* reveals that *GhPP2-33* regulates salt tolerance

**DOI:** 10.1186/s12864-023-09546-4

**Published:** 2023-08-18

**Authors:** Fei Wei, Pengyun Chen, Hongliang Jian, Xiaohao Guo, Xiaoyan Lv, Boying Lian, Mengxi Sun, Li An, Xinyu Dang, Miaoqian Yang, Hongmei Wu, Nan Zhang, Aimin Wu, Hantao Wang, Liang Ma, Xiaokang Fu, Jianhua Lu, Shuxun Yu, Hengling Wei

**Affiliations:** 1 Zhengzhou Research Base, National Key Laboratory of Cotton Bio-Breeding and Integrated Utilization, Zhengzhou University, Zhengzhou, 450001 China; 2 National Key Laboratory of Cotton Bio-Breeding and Integrated Utilization, Institute of Cotton Research of CAAS, Anyang, 455000 China

**Keywords:** *Gossypium hirsutum*, Phloem protein 2, Salt stress

## Abstract

**Background:**

Phloem protein 2 (PP2) proteins play a vital role in the Phloem-based defense (PBD) and participate in many abiotic and biotic stress. However, research on PP2 proteins in cotton is still lacking.

**Results:**

A total of 25, 23, 43, and 47 *PP2* genes were comprehensively identified and characterized in *G.arboretum*, *G.raimondii*, *G.barbadense*, and *G.hirsutum*. The whole genome duplication (WGD) and allopolyploidization events play essential roles in the expansion of PP2 genes. The promoter regions of *GhPP2* genes contain many *cis*-acting elements related to abiotic stress and the weighted gene co-expression network analysis (WGCNA) analysis displayed that *GhPP2s* could be related to salt stress. The qRT-PCR assays further confirmed that *GhPP2-33* could be dramatically upregulated during the salt treatment. And the virus-induced gene silencing (VIGS) experiment proved that the silencing of *GhPP2-33* could decrease salt tolerance.

**Conclusions:**

The results in this study not only offer new perspectives for understanding the evolution of *PP2* genes in cotton but also further explore their function under salt stress.

**Supplementary Information:**

The online version contains supplementary material available at 10.1186/s12864-023-09546-4.

## Background

The phloem is a bidirectional active transport system with a diverse array of molecular contents, and the phloem contains a large and diverse collection of molecule types involved in nutrient transport, inter-organ signaling, and adaptation to stress [1]. Phloem-based defense (PBD) is induced by multiple kinds of stresses, including wounding, oxidative conditions, and insect attacks [2–5]. Proposed components of PBD include the phloem protein 1 (PP1) and phloem protein 2 (PP2), which represent a type of the most abundant proteins in the phloem sap [6].

PP2 proteins play a vital role in the establishment of PBD. In previous studies, it was found that PP2 proteins could play a vital role in responding the biotic and abiotic stress. In *Arabidopsis*, the overexpression of *AtPP2-A1* could improve the resistance to the *Myzus persicae* [7]. After the mite feeding, the *AtPP2-A5* overexpressing lines showed less quantified damage while the *pp2a5* lines showed greater quantified damage [8]. The *AtPP2-B11* could play a positive and negative role in salt stress and drought stress, respectively [9, 10]. The further study found that *AtPP2-B11* could interact with *AtSnRK2.3* and degrade the *AtSnRK2.3* to play as a negative regulator in ABA signaling [11]. Similarly, the *AtPP2-B1* could also negatively regulates ABA signaling by taking part in the process of the downregulation of many ABA-inducible genes, such as *AtABI4* and *AtABI5* [12]. In *Cucumis sativus*, the overexpression of *CsPP2-A1* could play a positive role in preventing aphid attacks and enhancing salt tolerance [13, 14]. In *Solanum habrochaites*, the overexpression of *ShPP2-1* could decrease cold tolerance [15]. In *Brassica napus*, the *BnPP2-6* plays a positive role in regulating *Sclerotinia* disease resistance caused by *Sclerotinia sclerotiorum*. However, the knowledge related to the PP2 proteins in cotton is still limited.

The genus *Gossypium* contains 45 diploid species and 7 tetraploid species, it was found that all of the *Gossypium* species shared a whole genome duplication (WGD) event around 60 million years ago (MYA) compared with the *Theobroma cacao* [16]. In addition, more than 95% fiber production is produced by two cultivated tetraploids species *G. hirsutum* [(AD)1, 2n = 4x = 52], *G.barbadense* [(AD)2, 2n = 4x = 52], and it was hypothesized that they from allopolyploidization of *G.arboreum* (A2, 2n = 4x = 26) and *G.raimondii* (D5, 2n = 4x = 26) at approximately 1–1.5 MYA [16, 17]. The *Gossypium* species play an indispensable role in the textile industry because it could provide natural fibers to meet around 35% of the world’s annual fiber demand. However, the production and quality of cotton are critically limited by abiotic stress, thus it is essential to mine the genes with abiotic stress resistance functions. Fortunately, the increasingly releasing genome and transcriptome sequencing data in cotton made it possible to explore the genes with resistance to abiotic stress and their evolution progress [17–19].

In this study, for the first time, the *PP2* genes were systematically identified and characterized in *Gossypium* species, and the phylogenetic, gene structure, duplication events, selective pressure, and *cis*-elements analysis were comprehensively analyzed. The further evolution analysis showed that the *PP2* genes in *Gossypium* species mainly expanded by the WGD and allopolyploidization events, while their structure and function might largely conserve during these evolutionary processes. The weighted gene co-expression network analysis (WGCNA) and the quantitative RT-PCR (qRT-PCR) assays showed that many *GhPP2s* could be responding to salt stress. Based on the above analyses, we chose *GhPP2*-33 to perform the virus-induced gene silencing (VIGS) experiment to determine its function in the salt stress, we found that the *GhPP2-33*-silenced plants decreased the salinity tolerance compared with control plants. Together, these results would provide new insights into the evolutionary history and functions of *GhPP2* genes and lay benefit to the process of salt-resistant varieties in cotton breeding.

## Results

### Identification of PP2 proteins

A total of 25, 23, 43 and 47 *PP2* genes were identified in *G.arboretum* (A_2_, 2n = 2x = 26), *G.raimondii* (D_5_, 2n = 2x = 26), *G.barbadense* ((AD)_2_, 2n = 4x = 52) and *G.hirsutum* ((AD)_1_, 2n = 4x = 52), respectively (Table S1). The 138 identified cotton PP2 proteins ranged from 105 to 562 amino acids (aa) in length with an average of 283 aa, the molecular weight varied from 12.17 to 59.42 kDa, and theoretical isoelectric point (pI) ranged from 4.46 to 9.50. The grand average of hydropathy (GRAVY) in this family showed that all proteins had negative values (-0.72–0.02), indicating that all PP2 proteins were hydrophilic in cotton (Table S1).

### Phylogenetic analysis and structure analysis of PP2 proteins

**Fig. 1:**
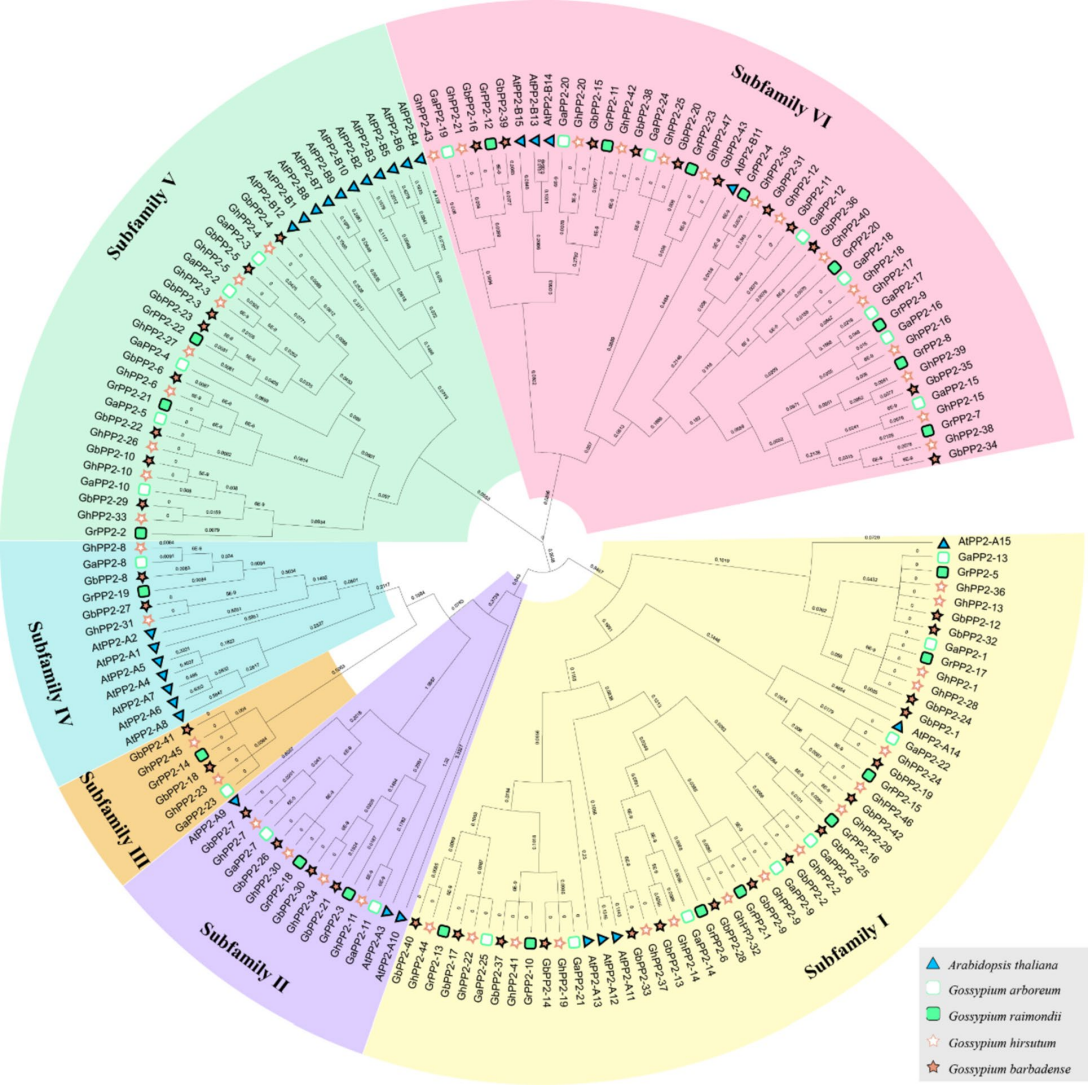
Phylogenetic analysis of predicted 168 PP2 proteins from five plant species. Each subfamily was shown in a different color. The prefixes At, Ga, Gr, Gh, and Gb are used to identify PP2 proteins from *A. thaliana*, *G. arboreum*, *G. hirsutum*, *G. raimondii*, and *G. barbadense*, respectively

Together with the *AtPP2s* obtained from the previous study, a phylogenetic tree including 168 PP2 proteins was generated. As clearly shown in the phylogenetic tree, these PP2 proteins can be divided into six distinct subfamilies, namely subfamily I-VI. The members in the subfamily I and subfamily VI constituted the largest number (31.36% and 27.21%), whereas the subfamily III had the lowest number (3.55%) and this subfamily might be specific to the *Gossypium* species (Fig. 1). Within each subfamily, the members from the *G. hirsutum* and *G. barbadense* cotton were almost twice that of the diploid cotton.

**Fig. 2:**
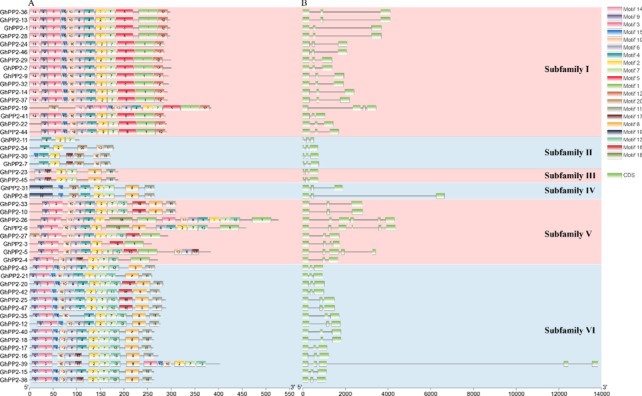
Gene structures and conserved motifs of PP2 members in *G. hirsutum*. **A** Distribution of conserved motifs in PP2 proteins. 20 putative motifs are indicated by colored boxes, and their motif name is displayed in the box in the upper right corner. **B** Exon/intron organization of *PP2* genes. Green boxes represent exons and black lines represent introns

The domain analysis showed that all PP2 proteins contain one or two PP2 domains located in the C-terminal. Besides the PP2 domains, F-box domains were found in the members of subfamily V and subfamily VI, respectively (Fig. S1). Most *GhPP2s* contain three exons, and only a few members harbored 4–6 exons (Fig. 2B). In addition, A total of 20 motifs were detected in *GhPP2* proteins, and the motif pattern was conserved in each subfamily. The motif 2, 4, and 7 were detected in most PP2 proteins. However, we also found that motif 14 and 5 only found in the subfamily I, and motif 19 exclusively belong to the subfamily IV (Fig. 2A). The gene structure and distribution of motifs varied in different subfamilies, while they were conserved in each subfamily (Fig. 2).

### Gene duplication and selection pressure of *PP2* genes

**Fig. 3:**
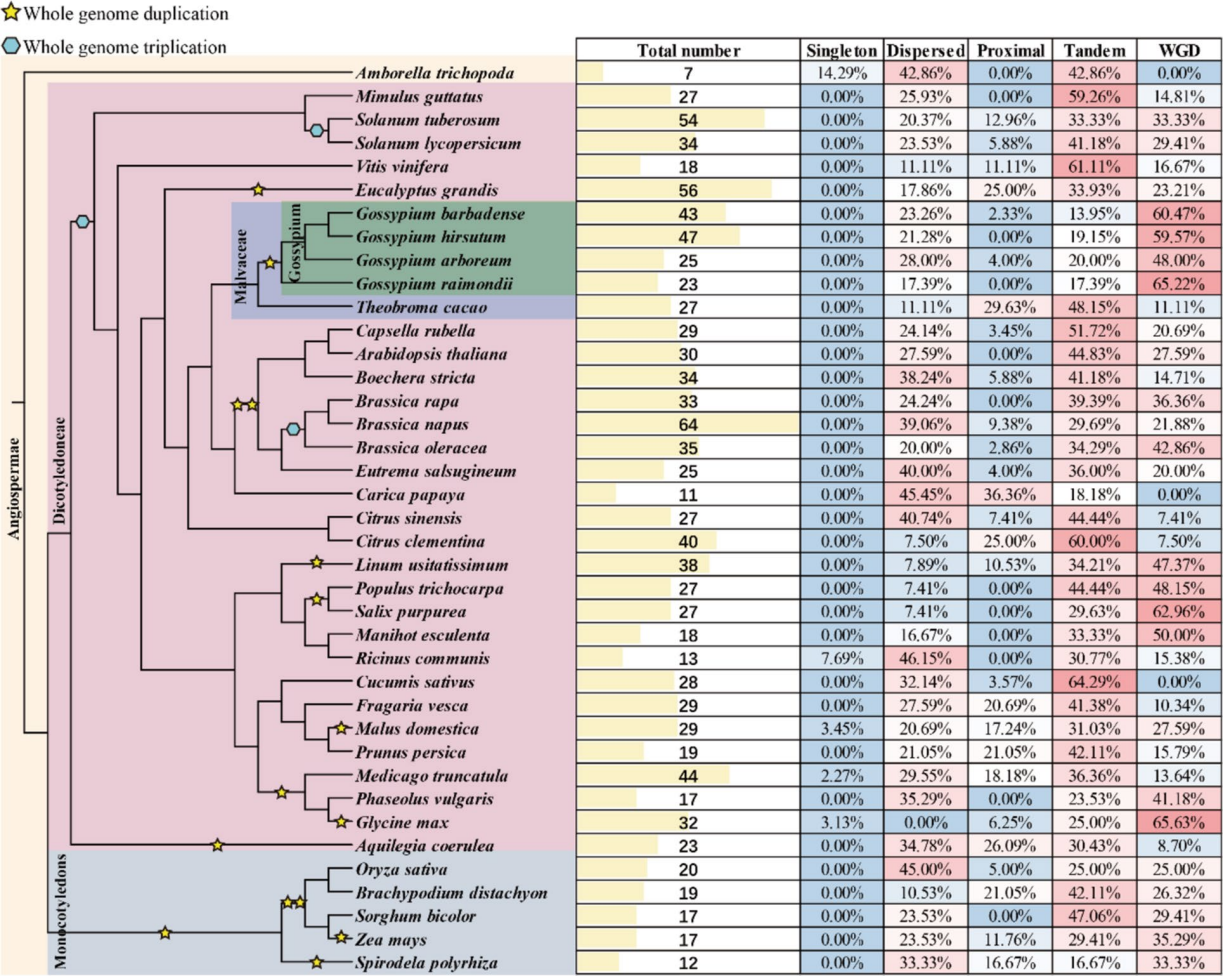
The number of identified *PP2* genes and the proportion of genes derived from different modes of duplication events in each plant species. The schematic diagram of the phylogeny of different plant species is retrieved from the TimeTree database (http://timetree.org/)

To further explore the expansion pattern during the evolution, we performed a comprehensive identification in 35 other plant species and identified a total of 1118 *PP2* genes in 39 species (Fig. 3). We found that *PP2* genes were widespread in monocotyledonous and dicotyledonous plants, and the number of *PP2* genes varied greatly in different plants. By performing McScanX, four duplication types were detected in cotton’s *PP2* genes, including WGD, tandem, dispersed, and transposed duplication events. Obviously, the WGD events played the main role in the expansion of *PP2* genes, followed by dispersed and tandem duplication. Similarly, the WGD is also the driving force in the expansion of *PP2* genes in *Salix purpurea*, *Glycine max*. However, in chosen species in this study (51.82%), *PP2* genes were mainly affected by the tandem duplication events, for instance, 64.29%, 61.11%, and 60.00% of *PP2* genes in *C.sativus*, *V.vinifera*, and *C.clementina* were expanded by tandem duplication. Additionally, dispersed duplication events are also an essential factor in expanding the *PP2* genes in many species, such as *C.papya* and *O.sativa*.

**Fig. 4:**
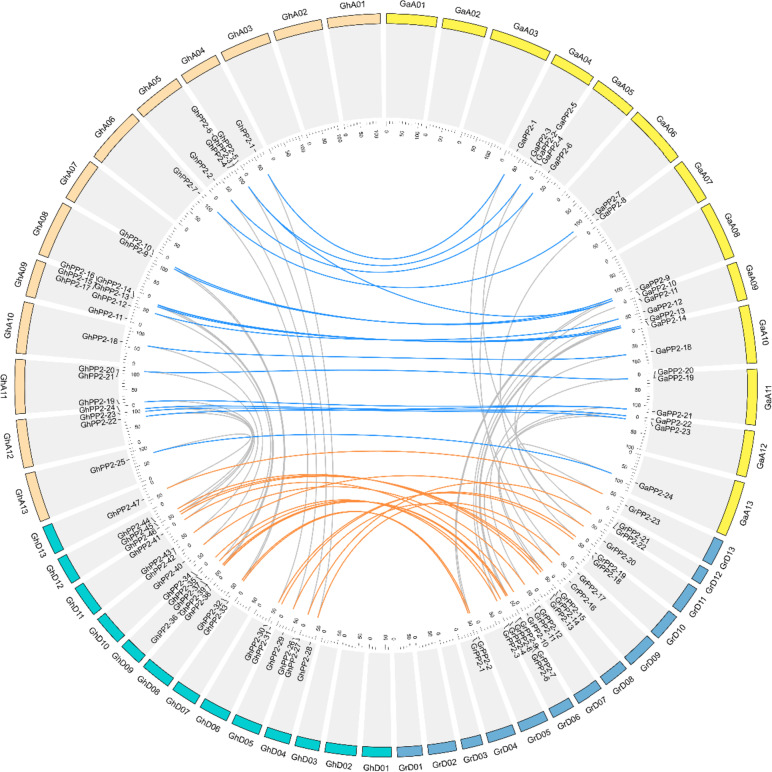
The chromosome distribution and collinearity analyses of *PP2* genes. GaA01-GaA13 and GrD01-GrD13 correspond to the chromosome of *G.arboreum* and *G.raimondii*, respectively; GhA01–GhA13, GhD01-GhD13 represent chromosomes of the At sub-genome, and the Dt sub-genome of *G. hirsutum*. The collinearity gene pairs between the allotetraploid and the diploid species are linked by blue and orange lines

For allotetraploid species *G.hirsutum* and *G.barbadense*, the *PP2* genes conserved well during the allotetraploidization event. *PP2* genes distribute on nine chromosomes in each *Gossypium* genome or subgenome, the distribution of PP2 in allotetraploid species is similar to their parental diploid species (Fig. 4, Fig. S2**)**. Moreover, the number of *PP2* genes in each allotetraploid is almost the sum of the two diploid species. Similarly, the number of *PP2* genes in *Brassica napus* was almost the sum of the two diploid species *Brassica oleracea* and *Brassica rapa* (Fig. 3). These results indicate that the allotetraploidization events also play a vital role in the expansion of the *PP2* genes, and they could be conserved during this evolution process.

We also analyzed the non-synonymous (Ka) to synonymous (Ks) substitution rates of *PP2* gene pairs detected in each diploid *Gossypium* species and each subgenome of allotetraploid species. All of the Ka/Ks ratios of gene pairs were lower than 1, which indicated that all the *PP2* genes might experience purifying selection pressure during evolution (Table S2).

### Analyses of *cis*-regulatory elements, expression profiles, and co-expression of *GhPP2s*

The 2000-bp upstream sequences of the *GhPP2s* were uploaded to the PlantCARE website to detect the *cis*-elements, many *cis-*elements were identified related to the stress response, light response, hormone response, growth and development were detected. Among these *cis*-elements, the MYB, MYC, and STRE elements were widely found in the promoter of *GhPP2s*, indicating that abiotic stress might induce the expression of *GhPP2* genes (Fig. S3).

A WGCNA analysis was employed to further explore the function of *GhPP2s*. The results showed that *GhPP2-33*, *GhPP2-32*, *GhPP2-41*, *GhPP2-44*, and *GhPP2-46* were detected in a network related to salt stress. Many transcription factors were also found in this network, such as *GhWRKY47* (GH_A11G1091)*, GhTFIIS* (GH_A09G2334)*,* and *GhMYB6* (GH_D05G2774), which suggests that many *GhPP2s* might co-expression with transcription factors to respond to salt stress (Fig. 6, Table S3).

Next, the qRT–PCR assays were performed to investigate the relative expression of *GhPP2*s at 0, 2, 4, 6, 8, 10, 12, and 24 h after the salt treatments. The results displayed that *GhPP2*s were upregulated after NaCl treatments at specific time points. For instance, *GhPP2-7* and *GhPP2-43* were upregulated after 1 h and 3 h salt treatment, while downregulated in the following hours. *GhPP2-15* was downregulated at 1 h-12 h while upregulated at 24 h. The *GhPP2-33* showed higher expression at all of the time points compared with the 0 h, especially the expression at 3 h and 24 h were 23–26 times higher than the 0 h (Fig. 6). These results indicate that *GhPP2s* could be involved in salt tolerance in plants, and we speculated that the *GhPP2-33* might play a vital role in responding to salt stress.

### Silencing of *GhPP2-33* attenuates salt tolerance in *Gossypium hirsutum*

Based on the above results, we select *GhPP2-33* as the candidate gene to perform the VIGS assay to measure its function in the salt treatment. The photobleaching phenotype of pTRV2:*PDS* plants and the qRT-PCR experiments confirmed the efficiency of the VIGS experiment (Fig. 7B and C). The pTRV2:*GhPP2-33* and pTRV2:00 plants showed similar phenotypes, and their MDA and proline content showed no significant difference before the salt treatment. However, after the 400mM NaCl treatment for two days, we observed that the pTRV2:*GhPP2-33* plants showed more wilt phenotype than the control plants (pTRV2:00) (Fig. 7A). Additionally, after the salt treatment, the content of malondialdehyde (MDA) and proline in pTRV2: *GhPP2-33* plants were noticeably higher and lower than the control plants, respectively (Fig. 7D and E). Therefore, the salt tolerance of the *GhPP2-33* silencing plant is weaker than the control plants, and we hypothesize that *GhPP2-33* could play a role in the salt stress tolerance.

## Discussion

The phloem-based defense (PBD) plays an essential role in various biological processes in the plant, and the PP2 proteins are one of the most critical factors in the PBD [2–5]. However, research on PP2 proteins in cotton is still lacking. In this study, the PP2 proteins were comprehensively characterized, including their phylogenetic relationship, gene expansion, selection pressure analysis, and expression analysis. Moreover, the WGCNA analysis displayed that the *GhPP2-33* might co-expression with many TFs and other *GhPP2s* in a network related to salt stress. The VIGS assays further confirmed that could play a positive role in responding to the salt stress in cotton. This study will provide essential information for further investigation of the function of cotton PP2 proteins.

### The evolution of *PP2* genes in cotton

In the present study, we identified *PP2* proteins in four cotton species and analyzed their properties. A total of 138 *PP2* genes were identified in different cotton varieties (*G.arboreum*, *G.raimondii*, *G.hirsutum*, and *G.barbadense*), and these genes could be classified into six subfamilies, the structure of *PP2* genes is relatively conserved in each subfamily (Figs. 1 and 2).

The *Gossypium* genus is a perfect model to research polyploidization [16, 20, 21]. In this study, many types of duplication events were detected in the expansion of *PP2* genes, and the WGD plays the most critical role in the expansion of *PP2* genes in *Gossypium* species. The tandem duplication plays a more vital role in the expansion of *PP2* genes in many species, such as *B.rapa* and *B.napus*, although the *Brassica* species experienced many rounds of WGD events [22]. Besides, the dispersed duplication event also plays an essential role in the expansion of *PP2* genes in many species, such as *C.papya* and *O.sativa* (Fig. 3). Therefore, the expansion pattern of *PP2* genes is very diverse in different species. In addition, the Ka/Ks ratio of gene pairs in *Gossypium* species generated by multiple duplication events was calculated, and all of the results were lower than 1, indicating that all of the PP2 members might underwent purifying selection pressure during evolution (Table S2).

On the other hand, in each allotetraploid cotton species *G.hirsutum* and *G.barbadense*, the number of identified *PP2* genes was almost the sum of the number of *PP2* genes in diploid cotton varieties *G.arboreum* and *G.raimondii* (Fig. 4 and Table S1). The *Brassica* species are also an ideal model in the research of WGD and allotetraploidization events [22]. Similarly, the number of *PP2* genes in allotetraploid species *B.napus* is almost the sum of diploid species *B.rapa* and *B.olerecea* (Fig. 3). Besides, the physical and chemical characteristics of the allotetraploid cotton were also similar with the diploid species (Table S1). These results showed that polyploidization events, including WGD and allotetraploidization events, play a significant role in the expansion of the PP2 gene family in *Gossypium* species, and strengthen the previous conclusion that the allotetraploid cotton species originated from the natural hybridization of the two diploid progenitors.

### The expression and function analysis of *GhPP2s*

Previous studies show that *PP2* genes play significant roles in response to salt stress in other plant species. For instance, *AtPP2-B11* could play a positive role in salt stress in *Arabidopsis* [10]. In *Cucumis sativus*, the overexpression of *CsPP2-A1* could play a positive role in preventing aphid attacks and enhancing salt tolerance [14].

**Fig. 5:**
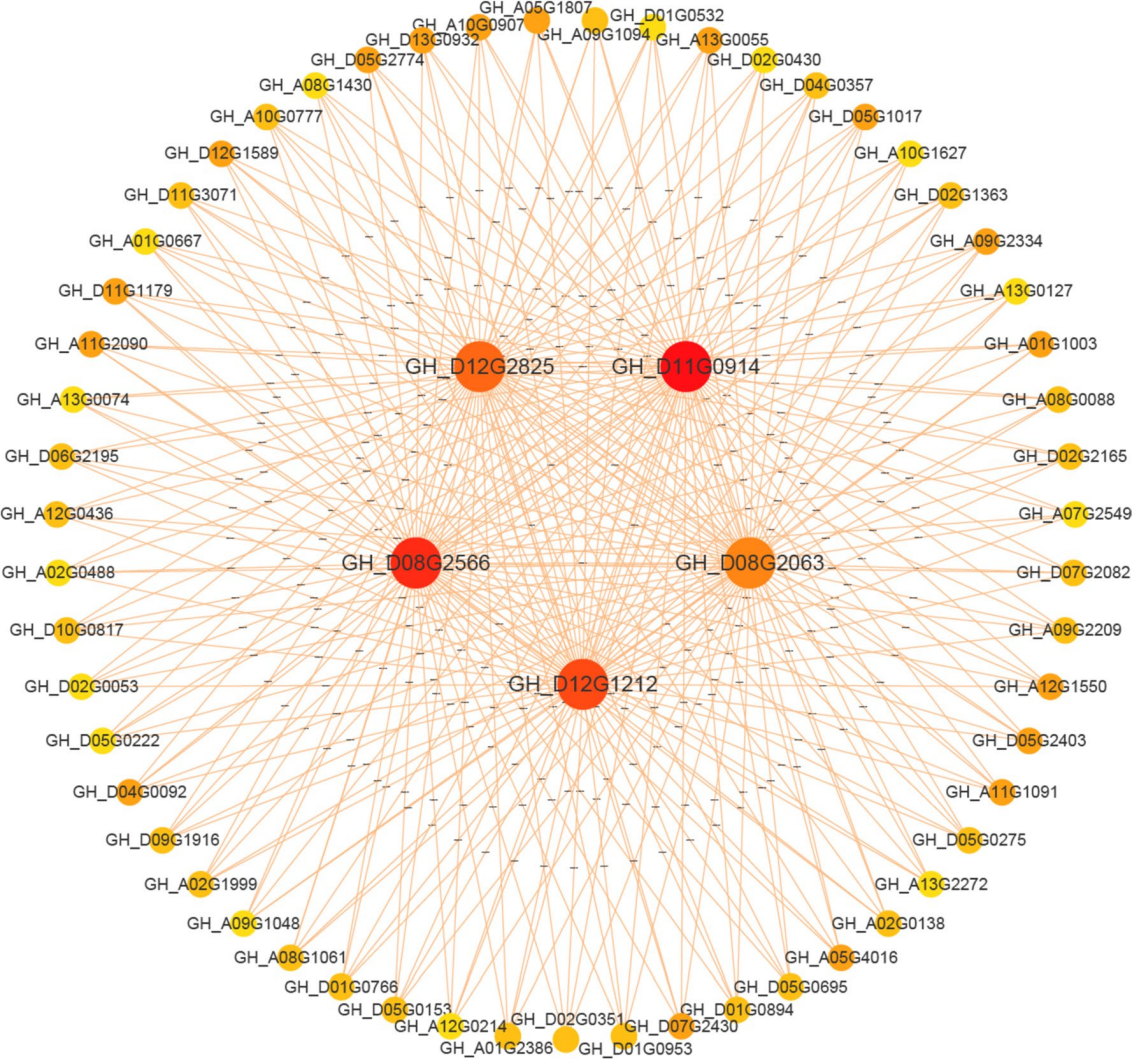
A co-expression network related to salt stress of *GhPP2-33* All the gene networks are constructed by the weighted gene co-expression network analysis (WGCNA), each node represents a gene. The information regarding genes displayed in the network is listed in the Table S3

**Fig. 6:**
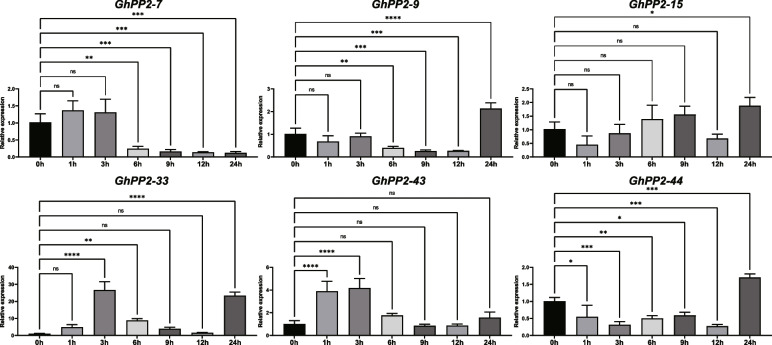
Expression patterns of *GhPP2s* under salt stress. Error bars represent the standard deviation of three independent biological replicates. (*p < 0.05; **p < 0.01; ***p < 0.001; ****p < 0.0001 when compared with the 0 h group)

In this study, many stress-responsive *cis*-elements for the plant were found in the promoter region of almost all PP2 genes (Fig. S3). Next, we further found that many *PP2* genes, such as *GhPP2-33*, were played as hub genes in a salt-stress-related co-expression network by performing WGCNA analysis (Fig. 5). And many TFs were also detected in this network, including the members from the WRKY, TFIIS, and MYB gene family, which might play a role in salt tolerance base on the previous studies [23–25]. In addition, by performing the qRT-PCR assays, many *GhPP2* genes could express higher under the salt stress, including *GhPP2-33* (Fig. 6). Thus, we speculate *GhPP2-33* might alter the salt stress in cotton.

**Fig. 7:**
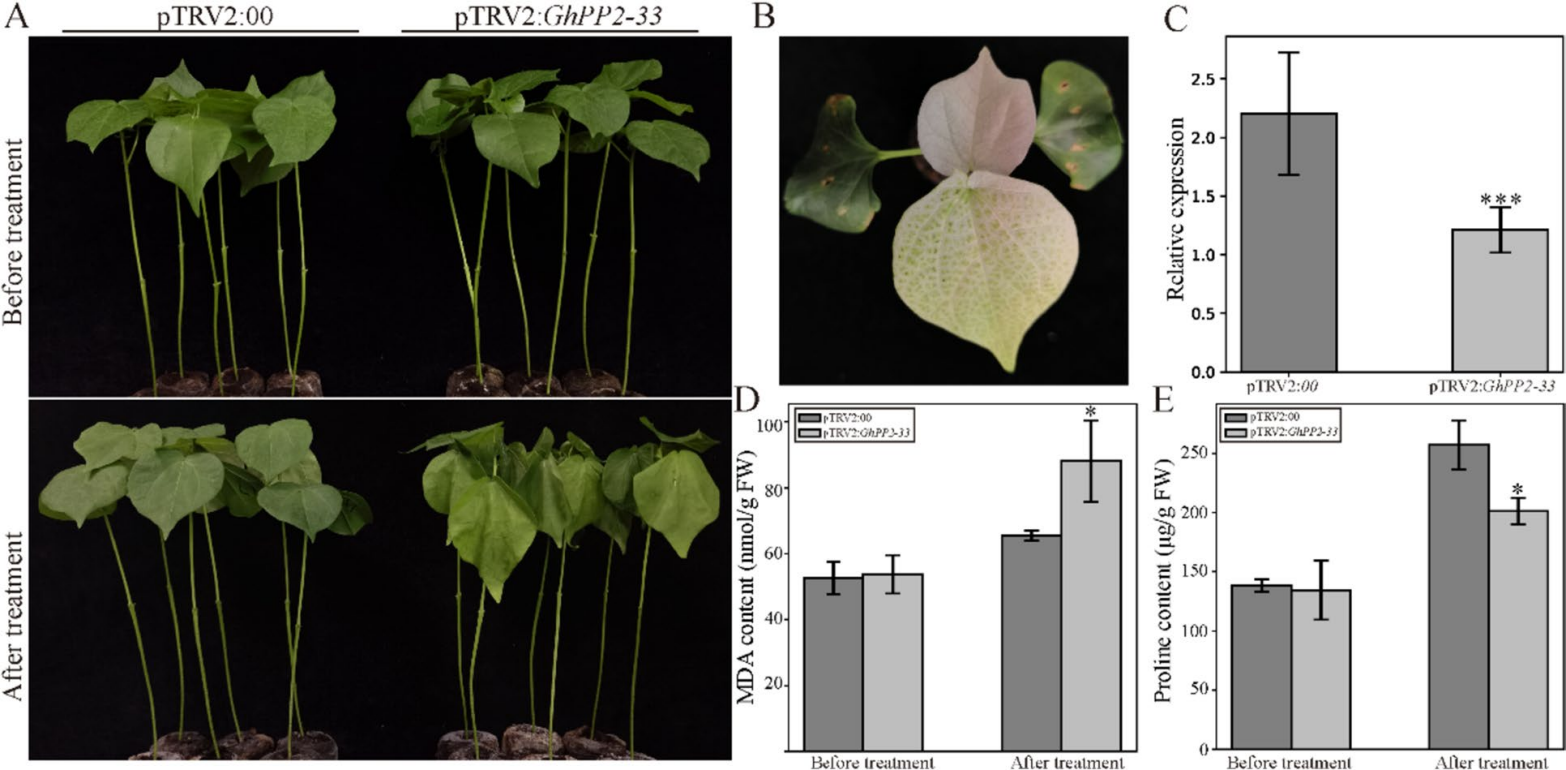
Silencing *GhPP2-33* via VIGS decreases salt tolerance in cotton. **A** Phenotype of pTRV2:*GhPP2-33* plants under 400 mM NaCl treatment. **B** Albino phenotype of pTRV2:PDS plants. **C** Relative expression of *GhPP2-33* in pTRV2:00 and silencing pTRV2:*GhPP2-33* plants via qRT-PCR analysis. **D** MDA contents. **E** Proline contents. Error bars represent the standard deviation of three independent biological replicates. (*p < 0.05; ***p < 0.001 when compared with the pTRV:00 group)

To further confirm the role of *GhPP2-33* in salt tolerance, we performed VIGS assays. After salt treatment, the silencing of *GhPP2-33* in cotton by VIGS experiment showed more wilted leaves in pTRV2:*GhPP2-33* plants than that in control plants (Fig. 7A). The measurement of MDA and proline are often used as the marker in displaying plant’s responses to abiotic and biotic stresses. MDA could represent the degree of lipid oxidative damage, while proline is a protective agent against osmotic stress [26, 27]. pTRV2:*GhPP2-33* plants possessed higher MDA, whereas the proline contents showed lower content than the control plant, suggesting that the *GhPP2-33* plays a positive role in responding to salt stress (Fig. 7D and E).

## Conclusions

In this study, a comprehensive analysis was performed to identify the PP2 proteins in the four *Gossypium* species. These PP2 proteins could be divided into six subfamilies, and the WGD and allotetraploidization events were the driving forces to expand the PP2 members in cotton. The analysis of *cis*-elements and the qRT-PCR experiments revealed that *GhPP2s* could participate in salt stress tolerance. Additionally, the WGCNA analysis revealed that *GhPP2-33* might play a role in salt stress tolerance. Finally, the VIGS assays displayed that the silencing of *GhPP2-33* could decrease salt tolerance. This study provides a comprehensive understanding of the PP2 proteins.

## Materials and methods

### The data retrieval and identification of PP2 proteins

A total of 39 species were chosen in this study, and the detail of their genome and annotation was listed in Table S4. By performing the hmmsearch with hidden markov model file of phloem protein 2 (PF14299.9) with the default parameter, the PP2 proteins were identified [28]. The protein sequences with the PP2 domain were further confirmed by the InterProscan program [29]. The protein properties of identified PP2 proteins were predicted by the ProtParam module in Biopython [30].

### Phylogenetic analyses

The identified PP2 proteins in *Arabidopsis thaliana* were obtained from a previous study [6]. Then, the PP2 proteins in *Gossypium* spp. and *Arabidopsis* were aligned using MAFFT (v7.310) [31]. Next, the BMGE was used to remove gaps in the alignment with the BLOSUM62 matrix and the gap rate cut-off of value is 50% [32]. The aligned protein was then used to construct a phylogenetic tree using the FastTree program with the LG model and finally visualized in evolview (http://www.evolgenius.info/evolview/) website [33, 34].

### Identification of gene collinearity and specific duplication events

To analyze gene collinearity, MCScanX software was performed to search all collinearity gene pairs between the different species and subgenomes with default parameters, and the circos (version 0.69–9) software was used to visualize the results [35, 36]. The duplication events were detected by the duplicate_gene_classifier program of the MCScanX software [35]. The gene pairs in the above duplication events were identified and classified by Dupgen_finder (https://github.com/qiao-xin/DupGen_finder) [37].

### The calculation of selective pressure

The detected gene pairs were further aligned by performing MAFFT software and formatted into an AXT format using the ParaAT pipeline [31, 38]. Next, the synonymous rate (Ks), nonsynonymous rate (Ka), and their ratio (Ka/Ks) of each gene pair were calculated by Kaks_calculator (v2.0) [39].

### The gene structure and conserved motifs analyses

The exon/intron position information in *Gossypium* spp. was extracted from the GFF/GTF files. In addition, the full-length protein sequences were submitted to the MEME website (http://meme.sdsc.edu/meme/itro.html) to detect motifs [40]. The results were displayed by TBtools software (version 0.1098765) [20].

### The identification of *cis*-elements

The 2000bp upstream genomic DNA sequences of *PP2* genes were submitted to the PlantCARE website (http://bioinformatics.psb.ugent.be/webtools/plantcare/html/) to predict the *cis*-acting elements [41].

### RNA-seq and WGCNA analysis

The transcriptome data were retrieved from a previous study (Accessions: PRJNA490626), which included seedlings treated with salt and drought at 1 h (hour), 3 h, 6 h, 12 h, and 24 h [17]. To generate clean reads, the raw RNA-seq reads were filtered by Trimmomatic with the default parameter (v0.3.9) [42]. Next, by performing HISAT2 (v2.1.0), the clean reads were mapped to reference genome (ZJU2.1) to produce SAM (Sequence Alignment/Map) format data and then converted to BAM (Binary Alignment/Map) format data using Samtools (v 1.9) [17, 43, 44]. The BAM files were assembled into transcripts and generated FPKM (Fragments Per Kilobase of transcript per Million mapped reads) using StringTie (v2.0) [45].

In this study, the genes with | logFC |> 1 and p-value 0.05 were chosen as DEGs and identified by edgeR (R version 3.10) [46]. Next, the weighted gene co-expression network, relevant modules, and hub genes were constructed by the WGCNA (version 1.69) package [47]. The weight value was calculated = pickSoftThreshold module in the WGCNA package, and β = 18 was chosen to execute power processing to obtain a scale-free adjacency matrix on the original scaled relationship matrix. The gene clustering and the module division were processed by the topological disparity matrix (dissTOM = 1-TOM) and the dynamic shearing algorithm, respectively. The minimum number of genes in the module was set as 30 (minModuleSize = 30), and the merge threshold of similar modules is 0.25 (cutHeight = 0.25). The final networks were visualized by Cytoscape software (v3.7.2) [47, 48].

### Plant cultivation, RNA isolation, and RT-qPCR analysis

The sterilized seeds of *G.hirsutum* L. (TM-1) were grown in an environment of 16/8 h day/night and 24/16 ◦ C. The three weeks old seedlings were uniformly selected and treated with 400 mM NaCl. Three biological replicates plant samples at 0, 1, 3, 6, 12, and 24 h were freshly collected and frozen in liquid nitrogen and stored at − 80 ◦ C till further analyses. The method of isolation of RNA, RNA reverse transcription, and the qRT-PCR experiments is as previously described [49, 50]. The results were analyzed with the 2^−∆∆^Ct method [51]. The primers used in RT-qPCR are listed in Table S5.

### Virus-induced gene silencing and stresses treatment

The genetic standard TM-1 cultivar was chosen for VIGS assays in this study. A 300bp fragment of *GhPP2-33* (291–590bp) was cloned into the pTRV2(pYL156) vector. The vectors pTRV2: 00, pTRV2:*GhPP2-33*, pTRV2: PDS, pTRV1 (pYL192, helper vector) were transformed into *A.tumefaciens* strain LBA4404. The experiments of the culture of *A.tumefaciens* and injection are the same as our previous studies [50, 52]. When the pTRV2: *PDS* plants showed an albino phenotype, the silencing efficiency of the pTRV2: *GhPP2-33* and pTRV2: 00 plants were further determined by qRT-PCR experiments. Next, the pTRV2: *GhPP2-33* and pTRV2: 00 plants were treated with 400mM salt for two days. The contents of MDA and Proline were determined using Malondialdehyde (MDA) Assay Kit and Proline Assay Kit according to the standard methods (Solarbio, Beijing, China), respectively.

### Supplementary Information

**Additional file 1:** **Table S1.** Basic information and biophysical properties of the predicted DUF4228 proteins in cotton.

**Additional file 2: Table S2.** The Ka and Ks values for duplicated gene pairs.

**Additional file 3: Table S3.** The detailed information on the co-expression genes of the WGCNA analysis in this study.

**Additional file 4: Table S4.** The plant genome annotation and their version used in this research.

**Additional file 5:** **Table S5.** List of primers used for qRT-PCR analysis.

**Additional file 6:** **Fig. S1.** Domain analysis of GhPP2 proteins. **Fig. S2.** The chromosome distribution and collinearity analyses of PP2 genes between G.barbadense and its parental diploid species. **Fig. S3.** cis-element analysis of the promoter of GhPP2s.

## Data Availability

Genome files of Arabidopsis thaliana, Oryza sativa, Gossypium species, and Brassica species were obtained from TAIR (https://www.arabidopsis.org/), Rice Genome Annotation Project (http://rice.uga.edu/), Cottongen (https://www.cottongen.org/), BRAD (https://brassicadb.org/) respectively. The genome files of the rest species mentioned in the article were retrieved from the Phytozome website (https://phytozome-next.jgi.doe.gov/). The RNA-seq data used in this study were retrieved from the NCBI Sequence Read Archive (SRA) database (https://www.ncbi.nlm.nih.gov/sra) under the accession code PRJNA490626.
