# Supplementary material for: A systematic analysis of the phloem protein 2 (PP2) proteins in Gossypium hirsutum reveals that GhPP2-33 regulates salt tolerance
Source: BMC Genomics. 2023 Aug 18;24:467. doi: 10.1186/s12864-023-09546-4 (PMC10439568; doi:10.1186/s12864-023-09546-4)
Supplement: Supplementary file 6 — Additional file 6: Fig. S1. Domain analysis of GhPP2 proteins. Fig. S2. The chromosome distribution and collinearity analyses of PP2 genes between G.barbadense and its parental diploid species. Fig. S3. cis-element analysis of the promoter of GhPP2s. [file 12864_2023_9546_MOESM6_ESM.docx]

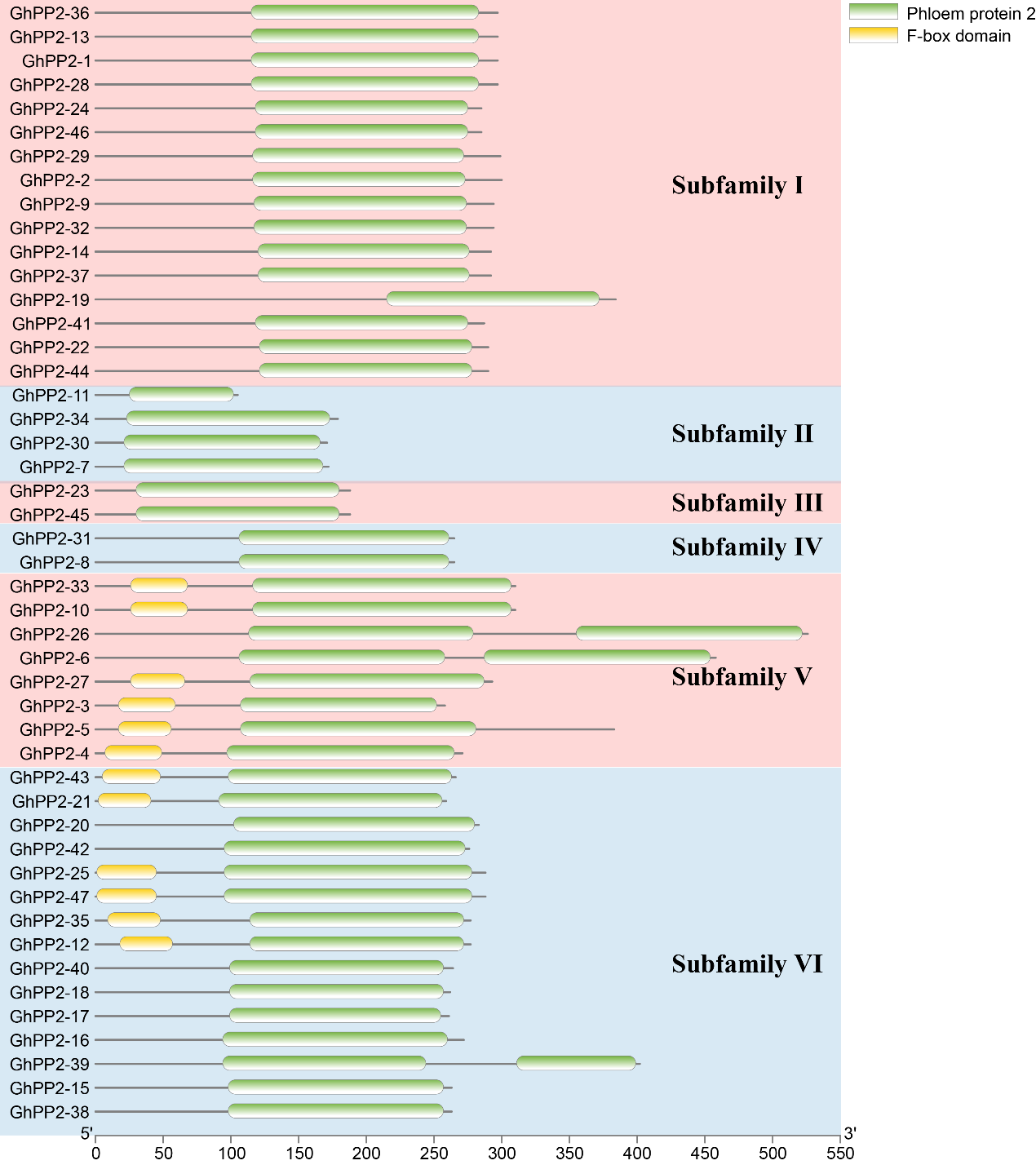


Fig. S1 Domain analysis of GhPP2 proteins.


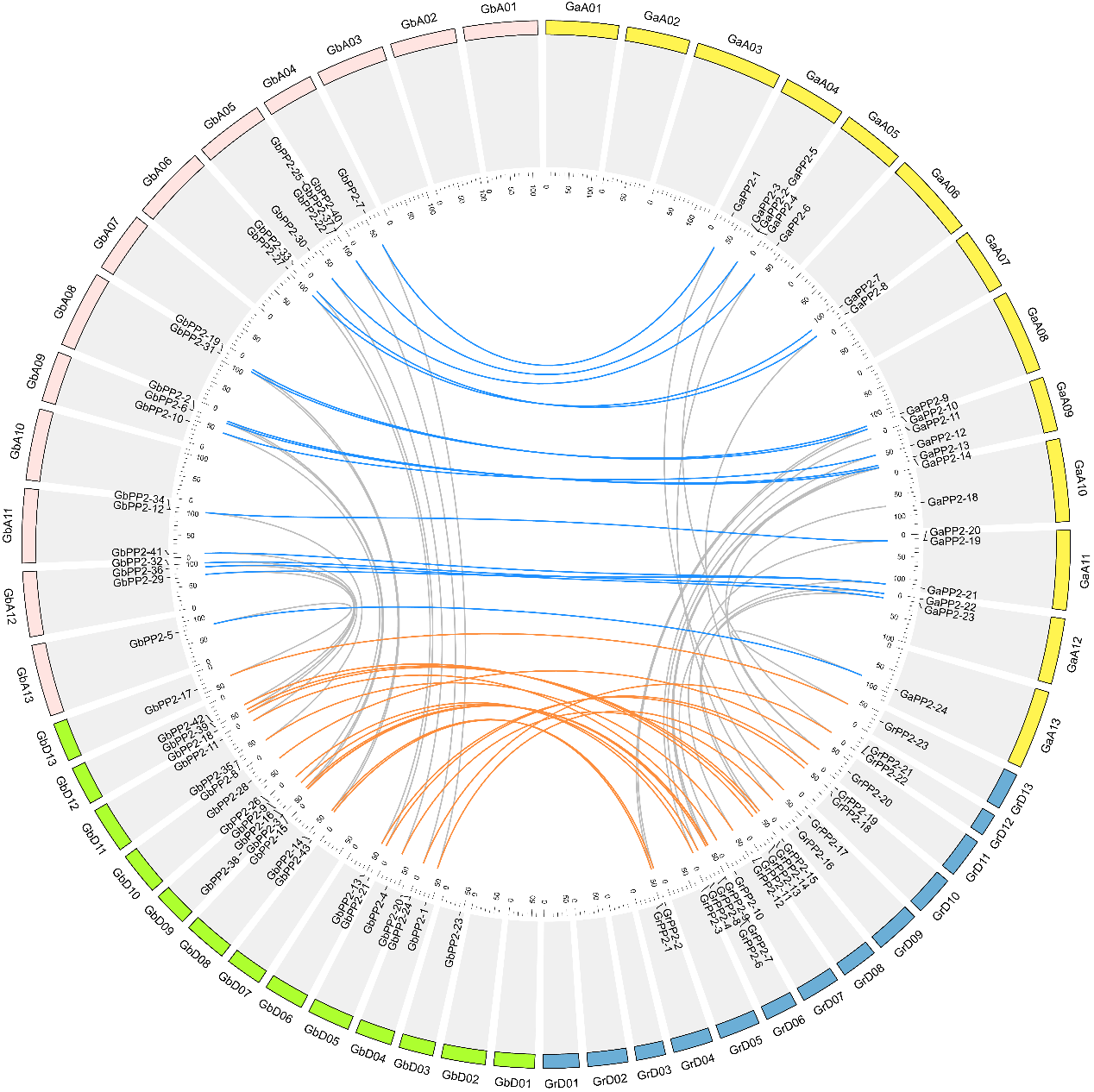


Fig. S2 The chromosome distribution and collinearity analyses of *PP2* genes between *G.barbadense* and its parental diploid species.


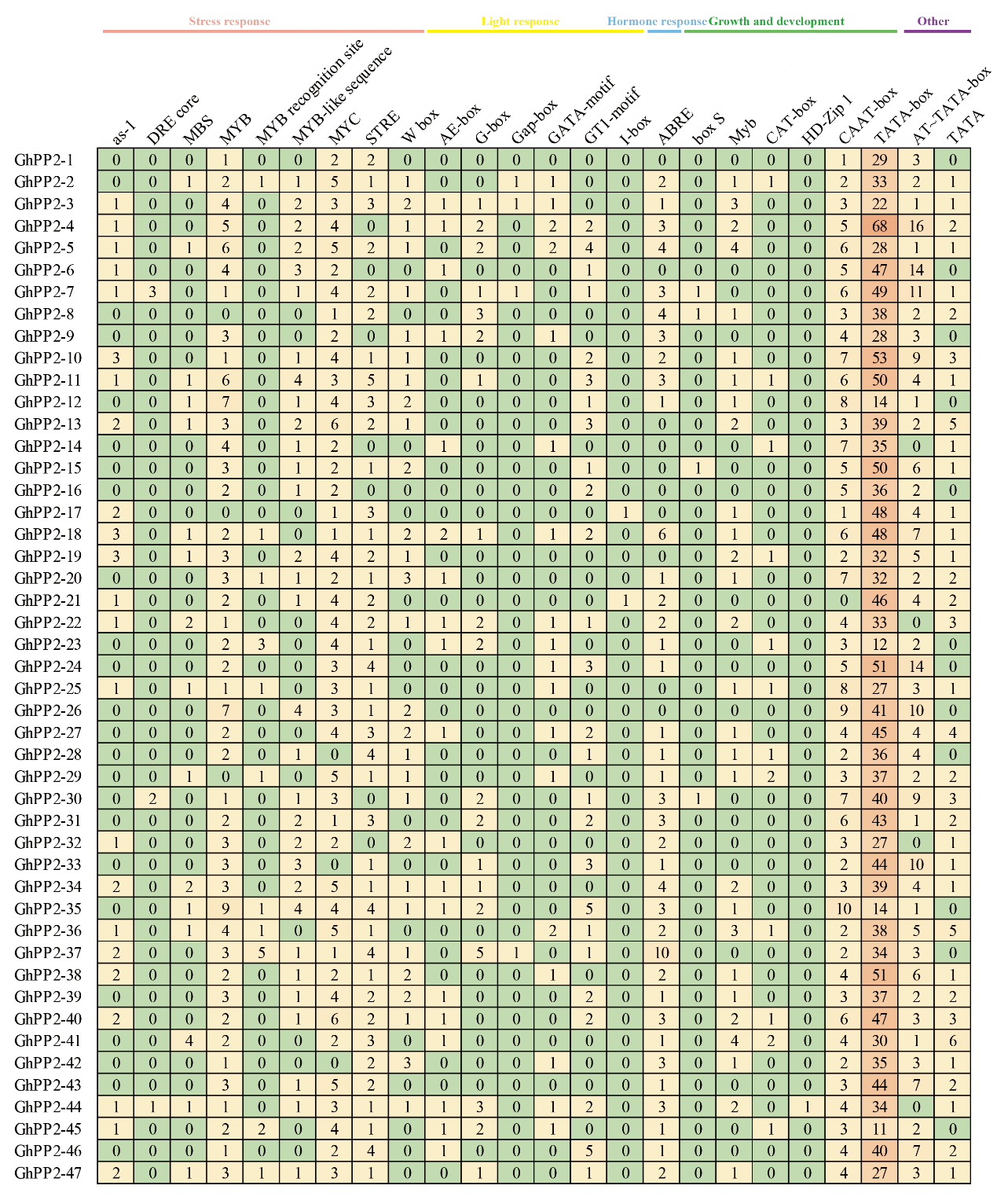


Fig. S3 *cis*-element analysis of the promoter of *GhPP2s*
